# Changes in the Comprehensiveness of Rural Medical Care for Older Japanese Patients during the COVID-19 Pandemic

**DOI:** 10.3390/ijerph182010772

**Published:** 2021-10-14

**Authors:** Ryuichi Ohta, Akinori Ueno, Chiaki Sano

**Affiliations:** 1 Community Care, Unnan City Hospital, Daito-cho Iida, Unnan 699-1221, Japan; 2 Unnan Public Health Center, Unnan 699-1311, Japan; ueno-akinori@pref.shimane.lg.jp; 3 Department of Community Medicine Management, Faculty of Medicine, Shimane University, Enya-cho, Izumo 693-8501, Japan; sanochi@med.shimane-u.ac.jp

**Keywords:** dependency, comprehensiveness, multimorbidity, rural medicine

## Abstract

Help-seeking behaviors (HSBs) refer to how people use lay and medical care to address their symptoms and diseases. The COVID-19 pandemic may have changed older, rural patients’ preferences and experiences regarding HSBs, thereby, affecting the comprehensiveness of medical support for communities. This study identified changes in the comprehensiveness of medical care for older, rural patients, who are often dependent on others for accessing medical services. This observational study was performed with patients who lived in Unnan City. Patients’ dependency and changes in comprehensiveness of medical services were assessed and calculated. The total usage of medical care decreased from 2018 to 2020 at all medical care levels. The proportion of patients who received comprehensive care was higher in 2020 than in 2018, at all care levels. At care dependent levels 3 to 5, the differences in the proportions were statistically significant. This study illustrates an association between the COVID-19 pandemic and the proportion of comprehensiveness of medical care among older rural patients with a decrease in medical care usage. Moreover, an improved proportion of comprehensiveness of medical care leads to appropriate HSBs. Going forward, HSBs and patient-centered care should be promoted by policy makers.

## 1. Introduction

The fragmentation of care, which refers to care received at more than one medical institution [1,2,3,4], is a critical issue when providing medical services and support to older patients who receive comprehensive care. In an aging society comprehensive care is required to holistically address various health problems and develop sustainable medical care systems in communities [5]. To ensure comprehensive care, approaches to multimorbidity, interprofessional collaboration (IPC), and community care must be enhanced. Regarding multimorbidity, as aging causes various health problems which can decrease the quality of life (QOL) [2], various healthcare professionals must collaborate to adequately/appropriately address these problems [1,3]. For older people to be able to continue living in their homes, medical professionals must comprehensively and effectively deal with multimorbidity, and care professionals need multiple skills to help take care of them. However, this can only be achieved through the cooperation of their families and social support systems [5]. The fragmentation of care can result in polypharmacy and increase complications due to multimorbidity, leading to mortality and decreased QOL among patients [2,3,4]. As medical science has advanced and specialized, an increasing number of patients are continuously being treated in multiple medical institutions, further accelerating the fragmentation of care. Fragmentation of care often occurs among older patients with multimorbidities [2,5]. As longevity has increased, a greater number of older patients have been receiving advanced care in specialized hospitals and lose their connections with primary care physicians [6,7]. Geographically, fragmentation of care can frequently occur in urban areas containing many multi-specialty medical institutions [8]. However, rural regions neighboring urban areas have been facing a challenge due to limited accessibility to urban medical institutions [9]. Fragmentation of care should be investigated not only in urban areas but also in rural areas to improve the proportion of comprehensiveness of medical care among rural communities.

The COVID-19 pandemic has changed the conditions of fragmentation of care and comprehensiveness of medical services. Difficulties associated with fragmentation of care among older patients are alleviated by caregivers [10,11]. Caregivers provide for older patients and enable them to visit hospitals multiple times [12]. However, the COVID-19 pandemic decreased caregivers’ accessibility due to various infection control measures, such as the implementation of lockdowns and a state of emergency in Japan [13,14]. Therefore, older patients may not have been able to access distant medical institutions, which may have changed their help-seeking behaviors (HSBs) [15,16]. Help seeking behaviors refer to how and where people use lay and professional care when they have symptoms and diseases [17]. The effective use of lay care can increase the appropriate usage of professional care. Using both lay and professional care in communities is essential for comprehensive care [9]. Additionally, preferences for lay and professional care are associated with perception of health conditions such as self-rated health and QOL [18,19]. Difficult circumstances regarding HSBs may be more prevalent in rural areas where many people live alone and have different social contexts compared to those who live in cities [20,21]. Recently, there has been an increase in the number of older people who need care, and an acceleration of fragmentation of care in rural areas [3,9]. The COVID-19 pandemic may have changed this trend and increased the challenges faced by older people. Moreover, the COVID-19 pandemic has reportedly changed social norms in rural areas [22]. Older, rural individuals may have faced social restrictions and limitations pertaining to their freedom of movement [22]. In addition, their places of HSBs can be directed toward primary care physicians, which could increase their usage of care within their communities, but not outside such communities [17]. However, regarding the place of HSBs, studies have not examined the changes related to where older, rural people receive medical care during the COVID-19 pandemic.

The pandemic’s effects on and potential mitigation of fragmentation of care in rural areas should be investigated to determine the current condition of the comprehensiveness of medical services. In rural areas, fragmentation of care depends on the availability and disparity of medical resources [9,10]. If a high proportion of dependent older patients visit hospitals in their communities during the COVID-19 pandemic, rural healthcare systems should be revised accordingly and be supported by general physicians who can manage multiple diseases [13,23]. Additionally, using information and communication technology (ICT) services and telemedicine can provide convenient online medical support, and reduce the risk of infections, for such patients [24,25]. Medical ICT applications in rural areas can improve the proportion of comprehensiveness of medical care [26,27]. Recognizing these changes and their relationship with fragmentation of care can lead to better rural medical care and awareness policies. In view of these motivations, this study investigated changes in the comprehensiveness of rural medical care during the COVID-19 pandemic.

## 2. Materials and Methods

This observational study analyzed dependent older patients living in Unnan City. In particular, we investigated changes in the comprehensiveness of medical care for these patients, based on their dependency pre- and post-COVID-19 (1 April to September 2018, and 1 April to September 2020, respectively).

### 2.1. Setting

Unnan City, located in southeast Shimane Prefecture, is one of the most rural areas in Japan. In 2020, its total population was 37,638 (18,145 males and 19,492 females), with 39% being over 65 years; this proportion is expected to reach 50% by 2025 [28]. Furthermore, Unnan has one of the lowest numbers of physicians per 1000 people in Japan [28]. It has 16 clinics, 12 home care stations, 3 visiting nurse stations, and 1 public hospital (Unnan City Hospital). Unnan City is adjacent to two urban areas: Matsue City and Izumo City. Matsue has 11 hospitals, 180 clinics, and 540 physicians, while Izumo has 11 hospitals, 144 clinics, and 811 physicians [9]. Unnan residents can reach Matsue’s and Izumo’s hospitals and clinics via a 30 min drive. As the Japanese medical system allows free access to medical institutions for all citizens, patients can choose any institution based on their needs and demands. Shimane Prefecture has developed a specialized, online ICT software called Mame-net, to share medical information and communicate with patients. It is currently used only by the local government of Shimane Prefecture, which has expanded its utilization. Using this system, clinics, hospitals, and nursing homes have been enabled to communicate with patients, and share medical and care information, such as acute and chronic changes, with patients as well as relevant healthcare professionals. After updating patients’ information via this system, notification emails without their precise information are sent automatically to all medical and care professionals in both institutions. This system could significantly drive interprofessional collaboration and improve palliative care in rural nursing homes [26,29]. During this pandemic, all healthcare facilities could use this system by applying to the Prefecture for its utilization.

### 2.2. Participants

All the patients from Unnan City who were certified as dependent on care and had accessed medical care between April and September of 2018 and 2020 were included in this study. The city’s population was 37,012 (48.0% were males) and 35,647 (48.0% were males) in 2018 and 2020, respectively. The number of people aged over 65 years was 14,437 and 14,367 in 2018 and 2020, respectively. The total number of dependent patients was 2192 (dependent care level 1: 480; level 2: 587; level 3: 392; level 4: 364; level 5: 369) and 2186 (dependent care level 1: 507; level 2: 515; level 3: 442; level 4: 375; level 5: 347) in 2018 and 2020, respectively.

### 2.3. Measurements

The demographics of the citizens of Unnan City were collected from the Unnan City citizen database. Anonymous patient data were extracted from the Shimane Prefecture insurance system. Data were collected between April 2018 and September 2020, comprising information about patients’ ages and sex; dependent care levels, based on the Japanese long-term insurance system (care level 1–5; 1: least dependent and 5: completely dependent); and institutions where they had received medical care [30]. The institutions were dichotomized as falling either inside or outside Unnan City. To measure the comprehensiveness of medical care, we used the proportion of comprehensiveness as the primary outcome based on a previous study [9]. The proportion of comprehensiveness was calculated as the number of patients who had received medical care in Unnan City, divided by the number of all patients who had done so. Patients who had received medical care in Unnan City and other places were counted in both categories. The proportion of comprehensiveness can demonstrate Unnan City’s capacity to provide comprehensive medical care to patients.

### 2.4. Analysis

The quantitative data were analyzed using Student’s t-test. Nonparametric data were analyzed using the Wilcoxon signed-rank test. Categorical data were compared using the chi-square test. Dependency and the proportion of comprehensiveness were also assessed and calculated. A significance level of *p* < 0.05, was used for all comparisons. Cases with missing data were excluded from the analysis. All statistical analyses were performed using EZR v1.50 (Saitama Medical Center, which is a graphical user interface for R (The R Foundation; http://www.r-project.org (access date: 10 August 2021) [31].

### 2.5. Ethical Considerations

Anonymity and confidentiality of patient information was ensured throughout the study. Only anonymous data were provided by the Unnan Public Health Center. Research information was posted on the hospital website without any identifying information. Contact information of the hospital representative was also listed on the website. All procedures included in this study were performed in compliance with the Declaration of Helsinki and its subsequent amendments. The Unnan City Hospital Clinical Ethics Committee approved the study protocol (No. 20200032).

## 3. Results

### 3.1. Participant Demographics

Table 1 shows the demographic data of care levels according to sex (men and women), age (80>, 80–84, 85–89, 90–94, and 95 ≤), and year (2018 and 2020). There were no statistical differences in care levels by sex or age between 2018 and 2020.

### 3.2. Change in Comprehensiveness of Medical Care

**Figure 1:**
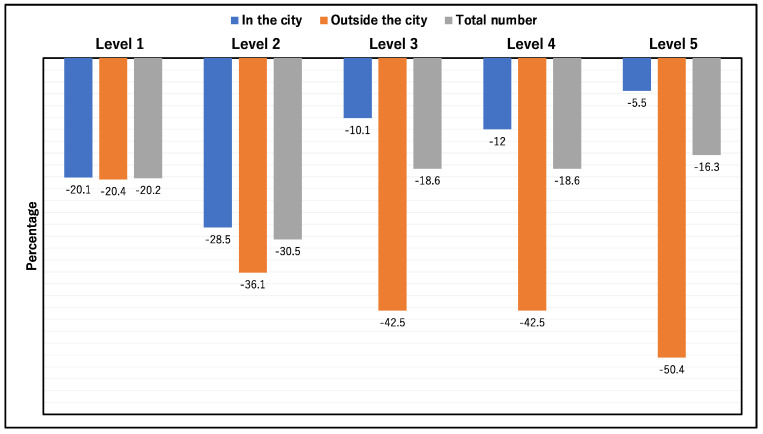
Reduction in the usage of medical care in care-dependent categories from 2018 to 2020.

The total utilization of medical care decreased from 2018 to 2020 at all care levels. The proportion of comprehensiveness was higher in 2020 than 2018 at all care levels. At care levels 3, 4, and 5, the differences between proportions in 2018 and 2020 were statistically significant (*p* = 0.020, 0.015, and 0.002, respectively) (Table 2). The medical care received in Unnan City has been reduced from −5.5% in 2018 to −28.5% in 2020. Meanwhile, the medical care received outside the city declined from −20.4% in 2018 to −50.4% in 2020. Overall, the proportions of utilization of medical care decreased during 2018–2020, from −15.1% to −30.5% (Figure 1).

## 4. Discussion

This study investigated the change in the comprehensiveness of the medical care in Unnan City during the COVID-19 pandemic. Compared to earlier, care-dependent older patients decreased their utilization of medical services during the pandemic. In addition, their patterns of using medical services as HSBs shifted from outside Unnan City to inside its rural areas. Furthermore, the use of medical care decreased during the pandemic—which, as long-term impacts of the pandemic, may help improve the quality of care and decrease the burden on rural healthcare services.

Improved proportions of the comprehensiveness of medical care during the pandemic could have been caused by various factors including a decrease in the usage of medical care. Government policies to contain the pandemic included restricting public movement to reduce the scope of infection transmission via human interaction, which could have decreased the usage of medical care [32]. As this study shows, such policies correlated with older patients’ use of local medical care within their communities, as opposed to those outside their communities. As dependent older patients would have been unable to visit healthcare facilities alone, they would have needed support from their families and community members [33,34]. However, restrictive policies against public movement would have made such assistance unlikely, prompting patients to rely on the facilities in their neighborhood. Such situations may have increased the proportion of the usage of rural healthcare facilities, as opposed to urban facilities; thus, increasing the proportion of comprehensiveness of medical care in rural areas. In addition, ICT usage is beneficial for increasing the scope and availability of medical services; our findings indicate that it might be practical in rural areas. Many medical facilities in urban areas have started using ICT to communicate with distant patients [35,36]. Metropolitan areas and hospitals might be best suited to use ICT to effectively prevent infections [37,38]. The lack of healthcare resources and older patients’ low familiarity with ICT could deter its usage in rural contexts [39,40], where direct communication with medical professionals is generally required for older patients. The COVID-19 pandemic has led to more healthcare professionals and older people using ICT to gain health-related information. Healthcare professionals could facilitate older patients’ utilization of ICT services, which would help mitigate healthcare professionals’ burden, leading to better healthcare [41].

During the COVID-19 pandemic, rural patients’ utilization of medical services decreased, which might be associated with their medical requirements, health conditions, or an increase in the proportion of comprehensiveness of medical care. In aging societies, frequent utilization of medical services can be a topic of concern [42]. Older people tend to demonstrate a variety of symptoms for which they require medical care. This can create a significant burden on themselves and their medical professionals [43]. The social distancing measures during the pandemic forced older people to stay by themselves and observe their symptoms, which may have affected their usage of medical care from rural facilities. These measures might have reduced the usage of medical care, which further lead to the increase in the proportion of comprehensiveness of medical care. However, even with decreased use of medical care, the mortality rate of care-dependent people in Japan was no higher than that in other countries [44]. Among older people’s HSBs, primary care utilization is the most common, followed by self-management, and consultation with their families and relatives [17]. During the pandemic, care-dependent patients may have reduced their primary care utilization and controlled their symptoms by themselves or received help from their families. The pandemic has been reported to have negatively affected the availability of healthcare services, and driven people to manage their symptoms using proper infection control methods [45,46,47]. The appropriateness of medical care utilization can differ across different medical systems. Future studies should investigate the relationship between changes in medical care utilization, HSBs, and health conditions during the COVID-19 pandemic.

The appropriate utilization of medical care can be encouraged during the COVID-19 pandemic. However, reducing such care may worsen the relationship between patients and healthcare professionals, which can diminish patients’ quality of life [48,49]. To sustain this relationship, hybrid consultation systems should be applied, using ICT to ensure the sustainability of patient-centered care and continuity of care by maintaining face to face interaction with the reduced risk of infection [50]. Furthermore, the present conditions of patients’ and healthcare workers’ medical actions should be sustained and enhanced through continuous provision of information and education about HSBs [17,19]. The appropriate utilization of medical care and the sustainability of patient-centered care can reduce the burden on healthcare professionals and improve the quality of care for patients during this pandemic.

One of the limitations of this study is its design. First, this observational study could not establish a cause-and-effect relationship between the COVID-19 pandemic and changes in medical care utilization. Although this relationship can be challenging to establish, this study supports the need for future investigations of this topic. Second, this study was conducted in a rural Japanese context. Therefore, it may not be generalizable to different, diverse sociocultural settings. However, as the pandemic had a global effect, the findings revealed in this study may apply to other rural areas, or less dense urban areas, in different parts of the world. Finally, this study’s observation period covered only one year, which may not suffice to determine long-term effects of the pandemic on HSBs. Future research should be conducted to investigate the pandemic’s impacts on HSBs in different sociocultural and geographical contexts, across different time periods.

## 5. Conclusions

This study demonstrated an association between the COVID-19 pandemic and the proportion of comprehensiveness of medical care among older rural patients with a decrease in medical care usage. The pandemic correlates with an improved proportion of comprehensiveness of medical care and an increase in the proportion of the utilization of healthcare facilities within the city, compared with facilities outside the city. This can lead to appropriate HSBs among older rural patients. Going forward, HSBs, ICT-driven and patient-centered care should be promoted by policy makers.

## Figures and Tables

**Table 1:** Participant demographics.

	Total	Care Level 1 (%)	Care Level 2 (%)	Care Level 3 (%)	Care Level 4 (%)	Care Level 5 (%)	*p*
Men							
2018	575	123 (21.4)	153 (26.6)	129 (22.4)	87 (15.1)	83 (14.4)	0.691
2020	437	96 (22.0)	103 (23.6)	94 (22.0)	78 (17.8)	66 (15.1)	
Women							
2018	1480	347 (23.4)	387 (26.1)	263 (17.8)	265 (17.9)	218 (14.7)	0.691
2020	1252	274 (21.9)	291 (23.3)	255 (20.4)	229 (18.3)	203 (13.4)	
Age							
80>							
2018	135	39 (28.9)	47 (34.8)	23 (17.0)	16 (11.9)	10 (7.4)	0.633
2020	84	18 (21.4)	29 (34.5)	17 (20.2)	10 (11.9)	10 (11.9)	
80–84							
2018	267	71 (26.6)	81 (30.3)	52 (19.5)	32 (12.0)	31 (11.6)	0.184
2020	130	46 (35.4)	31 (23.8)	18 (13.8)	14 (10.8)	20 (15.4)	
85–89							
2018	626	158 (25.2)	153 (24.4)	110 (17.6)	110 (17.6)	95 (15.2)	0.965
2020	495	121 (24.4)	117 (23.6)	87 (17.6)	87 (17.6)	83 (16.8)	
90–94							
2018	635	145 (22.8)	171 (26.9)	117 (18.4)	111 (17.5)	91 (14.3)	0.297
2020	594	132 (22.2)	131 (22.1)	126 (21.2)	112 (18.9)	93 (15.7)	
95≤							
2018	392	57 (14.5)	88 (22.4)	90 (23.0)	83 (21.2)	74 (18.9)	0.799
2020	387	53 (13.7)	86 (22.2)	101 (26.1)	84 (21.7)	63 (16.3)	

Care levels: dependent care levels, based on the Japanese long-term insurance system (care level 1–5; 1: least dependent and 5: completely dependent).

**Table 2:** Change in the proportion of comprehensiveness of medical care.

Care Level	In the City	Outside the City	Total Number	Proportion of Comprehensiveness	*p*
Level 1					
2018	427	176	603	0.708	1
2020	341	140	481	0.709	
Level 2					
2018	505	180	685	0.737	0.451
2020	361	115	476	0.758	
Level 3					
2018	355	128	483	0.735	0.020
2020	319	78	410	0.778	
Level 4					
2018	316	120	436	0.725	0.015
2020	278	69	355	0.783	
Level 5					
2018	271	85	356	0.761	0.002
2020	256	42	298	0.859	

Note: The proportion of comprehensiveness is equivalent to the number of patients who received medical care in Unnan City divided by the total number of patients who sought medical help [9].

## Data Availability

The datasets used and/or analyzed during the current study may be obtained from the corresponding author upon reasonable request.
